# SYNJ2BP inhibits tumor growth and metastasis by activating DLL4 pathway in hepatocellular carcinoma

**DOI:** 10.1186/s13046-016-0385-0

**Published:** 2016-07-20

**Authors:** Xiao Liu, Jiangjiao Zhou, Ning Zhou, Jianwei Zhu, Yong Feng, Xiongying Miao

**Affiliations:** Department of General Surgery, The Second Xiangya Hospital, Central South University, Changsha, Hunan 410011 China; Hepatobiliary Surgery Department, Hunan Provincial People’s Hospital, Changsha, Hunan 410005 China

**Keywords:** Hepatocelluar carcinoma, SYNJ2BP, Prognosis, Metastasis, DLL4

## Abstract

**Background:**

Synaptojanin 2 Binding Protein (SYNJ2BP) is essential to cell proliferation. Previous studies show that SYNJ2BP participates in sprouting angiogenesis, which plays an important part in several abnormal conditions including cancer. However, the activity of SYNJ2BP in hepatocellular carcinoma (HCC) has not been elucidated yet.

**Methods:**

Firstly, real-time PCR and western blotting (WB) were adopted to evaluate SYNJ2BP expressions in HCC tissues and HCC cell lines. Secondly, we did follow-up and prognostic study to explore the association of SYNJ2BP expression and HCC patients prognosis. Thirdly, we induced or silenced SYNJ2BP expression on selected HCC cell lines and explored the function of SYNJ2BP *in vitro* and *in vivo*. Lastly, we conducted Cignal Finder Cancer 10-Pathway Reporter Array in combination with loss- and gain-of-function assay to investigate potential mechanisms.

**Results:**

Through various techniques we found that SYNJ2BP was decreased in HCC tissues and HCC cell lines. The subsequent analysis showed that low expression of SYNJ2BP was associated with tumor size, tumor nodule number, vascular invasion, TNM stage and BCLC stage, and was an independent risk factor for survival of HCC. Later, the *in vitro* experiments demonstrated that SYNJ2BP inhibited HCC cells invasion, migration and proliferation, also the *in vivo* testing revealed that SYNJ2BP inhibited tumor growth and metastasis. Finally, we also uncovered that SYNJ2BP inhibited HCC growth and metastasis through activating DLL4-mediated Notch signaling pathway.

**Conclusions:**

Collectively, our data provide evidence that SYNJ2BP may act as a tumor suppressor during HCC development and could serve as a potential therapeutic target.

## Background

Hepatocellular carcinoma (HCC) is one of the most common cancers worldwide and ranks as the second-leading cause of cancer-induced death in men [1]. Especially for our country with a high incidence of hepatitis B, the magnitude of the problem should never be underestimated [2]. With the development of modern medicine, many technologies have been adopted in treatment of HCC [3]. So far surgical resection is still the major strategy for HCC, but the survival remains disappointing because of high rates of intrahepatic and extrahepatic metastasis [4]. For that it seems reasonable to confirm early detection and take timely treatment. Over the past decades many molecular biomarkers involved in HCC have been identified [5–8], and also researches on microRNAs [9], lncRNA [10, 11] and inflammatory factors [12] have attracted extensive attention to scientists. However molecular mechanisms of HCC development are not fully understood, to further elucidate the mechanisms of HCC and explore effective treatment is of paramount importance.

Synaptojanin 2 Binding Protein (SYNJ2BP) is a protein (145 amino acids) coding gene located at 14q24.2 and can regulate localization of Synaptojanin-2 and endocytosis of Activin type II receptors [13, 14]. There have been studies showing that SYNJ2BP protein has a single PDZ domain and is widely expressed in vertebrates [13, 15]. Besides, Adam MG, etal. proved that SYNJ2BP stabilizes Notch ligands and inhibits sprouting angiogenesis [16]. Considering Notch signaling participates in vascular remodeling [17, 18] which plays an important part during cancer progression [19, 20]. There’s been clear evidence showing that Notch signaling goes hand in hand with occurrence and development of many cancers [21, 22]. Existing studies also show that Notch signaling plays a multifaceted role in cancer, and in some contex Notch serves as a suppressor during HCC development [23, 24]. We also searched information with the aid of bioinformatics, results from ‘The Cancer Genome Atlas’ (TCGA) showed that a RNA-Seq screening containing 423 samples showed that the relative expression of SYNJ2BP in HCC tissues is obviously lower than that of peritumoral tissues (PTs) (Supplementary), which is in accordance to our verification. Altogether, there are good reasons to hypothesize that SYNJ2BP may play a role in HCC progression.

By far, there has been no research about SYNJ2BP function in HCC yet, so we conducted this study to explore potential role of SYNJ2BP in HCC. First SYNJ2BP expression in human HCC tissues and cell lines were determined, and then SYNJ2BP function was tested *in vitro* and *in vivo*. Finally, we investigated the potential molecular mechanisms of SYNJ2BP in HCC development.

## Methods

### Tissue specimens and patients

Patients and specimens in our study were derived from the Second Xiangya Hospital of Central South University, we selected 98 patients from 450 cases from January 2007 to June 2010. All the patients received hepatectomy and no one received neoadjuvant therapies. Among these patients, 28 matched fresh tumor tissues, peritumoral tissues (PTs) and 4 fresh normal live tissues (NLs) derived from hemangioma of liver were selectively chosen. Tumor tissues were collected from tumor nest, while PTs were obtained surrounding the tumor tissues (1 cm from tumor margin). All the tissues above were routinely snap-frozen in liquid nitrogen and stored at 280 °C for real-time quantitative reverse-transcription polymerase chain reaction (qRT-PCR) and western blot analysis. The samples employed for immunohistochemistry (IHC) were paraffin embedded. Histopathology was evaluated by two certified pathologists at the Department of Pathology, the second Xiangya Hospital of Central South University. In the part of observational study, written informed consent was obtained from all patients before surgery. All human materials were obtained with informed concent from participants and approved by the Ethics Committee of the Second Xiangya Hospital of Central South University.

### RNA isolation, quantitative real-time reverse transcription polymerase chain reaction (qRT-PCR)

Total RNA was extracted from cell lines or frozen tissues using Trizol reagent (Invitrogen, Carlsbad, Calif) according to the manufacturer’s protocol. Quantitative Real-time PCR was performed using the SYBR Green Real-time PCR Master Mix (Toyobo, Osaka, Japan) as described. The primer sequences were as follows: for real‐time PCR: SYNJ2BP forward, 5′-CTGCACCAGGATGCTGTAGA-3′, SYNJ2BP reverse, 5′-TGGCACCAGCACCATAAATA-3′; GAPDH was used as an internal control using the following primers: GAPDH forward, 5′-GCACCGTCAAGGCTGAGAAC-3′,GAPDH reverse, 5′-TGGTGAAGACGCCAGTGGA-3′. All the primers above were bought from Sangon Biotech.The experiments were done in triplicates.

### Western blotting

Total protein was extracted using RIPA lysis buffer supplemented with 1 % Phenylmethanesulfonyl (PMSF) and separated by sodium dodecyl sulfate-polyacrylamide gel electrophoresis (SDS-PAGE) and then transferred onto polyvinylidenefluoride (PVDF) membranes (Millipore, Bedford, Mass). The blotted membranes were incubated with appropriate antibody at optimal dilution. Finally, the blots were developed using enhanced SuperSignal West Pico chemiluminescence (Pierce Rockford USA). The SYNJ2BP antibody was obtained from Proteintech group (Proteintech, USA), corresponding secondary antibodies and the other proteins were purchased from Santa Cruz Biotechnology (Santa Cruz, CA).β-actin protein was also determined by using the specific antibody (Santa Cruz, CA) as a loading control. All experiments were carried out in triplicates.

### Immunohistochemistry

Formalin-fixed paraffin sections corresponding to previous 98 patients were gained, sections were stained for SYNJ2BP using the streptavidin-peroxidase system (Zhong-shan Goldenbridge Biotechnology, Beijing, China). Negative control slides were probed with goat serum followed by the secondary antibody under the same conditions. The expression level of SYNJ2BP was scored using the Shimizu [25] criteria. First scoring was obtained according to percentage of positive hepatocytes: 0, ≤10 % positive; 1+, 11 % to 25 % positive; 2+, 26 % to 50 % positive; 3+, 51 % positive. Secondly, SYNJ2BP expression was scored 0, and 1+, 2+, and 3+ according to expression intensity. Ultimately, we added the scores above, and SYNJ2BP expression in HCC specimens was divided into a low-expression group (0 or 1+) and a high-expression group (2+ or 3+).

### Follow-up and prognostic study

Our team conducted the follow-up part through telephone or visiting regularly, recurrence or metastasis were monitored by clinical examination, alpha-fetoprotein levels, ultrasonography,high-resolution contrast-enhanced CT or magnetic resonance imaging (MRI) tests etc. Every 3 months in the first three years after operation and twice a year after that are recommended. The follow-up began the date of operation and ended the date of death or last follow-up. Deaths from other causes were treated as censored cases. The period from hepatectomy to the signs of recurrence was defined as disease-free survival (DFS).

### Cell lines and cell culture

In this study, we adopted cell lines as follows: L02 (normal liver cell line), Hep3B, HepG2, PLC/PRF/5, SMMC-7721 and HCCLM3 which are commonly used in HCC research. Among the cells above: L02 cells were obtained from the Tumor Institute of Central South University, Changsha, China; Hep3B, HepG2, PLC/PRF/5, SMMC-7721 and HCCLM3 cell lines were kindly gifted by the Shanghai Institutes for Biological Sciences of the Chinese Academy of Sciences. All cells were maintained in Dulbecco’s modified Eagle medium(GIBCO, Grand Island, NY) supplemented with 10 % fetal bovine serum (GIBCO) and 1 % antibiotics at 37°Cwith 5 % CO2.

### Vector construction and transfection

According to SYNJ2BP expression profile, we chose Hep3B and HCCLM3 cells for further investigation. All the ectopic expression and knockdown lentiviruses as well as their negative control (NC) lentiviruses were bought from GeneChem (Shanghai,China). Then the plasmids were transfected into corresponding cells. The ectopic expression sequences for SYNJ2BP were: SYNJ2BP-forward:5′-CCGCTCGAGATGGACTACAAAGACGATGACGACAAGAACGGAAGAGTG-3′, SYNJ2BP reverse:5′-CGGGATCCTCAAAGTTGTTGCCGGTA-3′. The 3 candidate hairpin sequences for SYNJ2BP were:sequence-1:sense,5′-CACCGGACGTAGATGCCACTGTCGT-3′,antisense,5′-AAACACGACAGTGGCATCTACGTCC-3′;sequence-2:sense,5′-CACCGGTAGACCTCTTTCGTAATGC-3′,antisense,5′-AAACGCATTACGAAAGAGGTCTACC-3′;sequence-3sense,5′-CACCGCAGTATGTCTCCAACGACAG-3′,antisense,5′-AAACCTGTCGTTGGAGACATACTGC-3′.The NC sequences for SYNJ2BP were: sense,5′-TTCTCCGAACGTGTCACGT-3′,antisense,5′-ACGTGACACGTTCGGAGAA-3′. Finally, qRT-PCR and Western Blot were carried out to validation the transfection efficacy. All assays were carried out in triplicates.

### Wound healing and transwell

One day before the wound healing assay, HCC cells were seeded into 35 mm dishes. When cellular density reached nearly 100 % confluence, we made a scraped line using a small pipette tip, and then put the cells back to incubator. In order to assess the rate of closure, micrographs were taken every 24 hours using an inverted microscope IX51 (OLYMPUS) [26]. Transwell assay was used to assess cell invasive ability [27]. A transwell dish has two chambers, we added medium containing 10 % fetal bovine serum (GIBCO) to the chamber below, while medium containing 0.1 % fetal bovine serum (GIBCO) to the upper chamber. 1 × 10^5^ cells were seeded into the upper chamber precoated with matrigel (BD Biosciences, Franklin Lakes, NJ) and cultured at 37 °Cwith 5 % CO2. 48 hours later, we removed gel and cells in the upper chamber and stained cells below the membrane with crystal violet (Beyotime Institute of Biotechnology). We managed to capture the cells with inverted microscope (OLYMPUS, IX51, Japan) and counted cells having got through the membrane. All assays were carried out in triplicates.

### MTT and colony formation

The MTT and colony formation assays were adopted to assess cell proliferation ability. For MTT assay, after digestion, 4000 cells together with 200 μl fresh medium were seeded into each well of 96-well plates, wells merely containing medium were used as negative control, and then all plates were routinely put into incubator for seven days. From the second day, we took six wells from each group for detection every day. 100 μl fresh medium containing MTT (5 mg/ml) was put into each well and incubated at 37 °C for 4 hours, then the medium was replaced by 150 μl DMSO and shaken at room temperature for 10mins. The absorbance was measured at 570 nm wavelength [28]. For colony formation assays, 500cells together with 2000 μl fresh medium were seeded into each well on 6-well plates (NEST Biotechnology Co.LTD.). Then cells were incubated at 37 °C with 5 % CO2 atmosphere. Two weeks later, medium was removed and cells were stained with crystal violet (Beyotime Institute of Biotechnology, Beijin, China) after washed. We captured the dishes with a camera (Canon, EOS 760D, Japan) and counted colonies which were bigger than 40um in diameter [29]. All assays were carried out in triplicates.

### Immunofluorescence

We also adopted immunofluorescence staining to analyze SYNJ2BP influence on cell migration. The sterile coverslips were laid on 6-well plates, each well with 1 × 10^5^ cells, then plates were incubated at 37 °C with 5 % CO2 atmosphere overnight. The next day, Phalloidin (SIGMA, Louis MO, USA) was used to stain F-actin filaments (red) of cells fixed on coverslips; 4, 6-diamidino-2-phenylindole (DAPI) was adopted to stain cell nuclei (blue). Then cell captures were obtained with the aid of inverted microscope (OLYMPUS, IX51, Japan). All assays were carried out in triplicates.

### Cignal finder cancer 10-pathway reporter array

The Cignal Finder Cancer 10-Pathway Reporter Array (Qiagen, Dusseldorf, Germany) was performed as described to pinpoint pathways regulated by SYNJ2BP. After preparation of complex formation according to the instruction, suspended cells were obtained from exponential phase of growth cells (1 × 10^6^/ml, 50 μl/well). Then the 96-well plates were put at 37 °C with 5 % CO2 atmosphere, 6 hours later previous medium was replaced with medium containing 10 % fetal bovine serum without antibiotics. The next day, complete medium were added and the cells were cultured for another 48 hours. Finally, the luciferase assay was carried out using the Dual-Luciferase Reporter Assay System (Promega, WI, USA).

### HCC mouse model

According to experimental design, we established model in BALC/c nude mice as described [30]. First we chose 4-week old male mice and randomly assigned them to groups; and then 5 × 10^6^ HCC cells were subcutaneously injected to the left upper regions of nude mice respectively; tumor diameter was measured using vernier calipers every 3 days for 4 weeks. Four weeks later, tumors were harvested and volume was calculated using the formula: (Length × Width^2^)/2 [30], meanwhile, tumors were also cut into pieces(1.0 mm^3^).And then we implanted those pieces under live capsule to mimic the primary HCC. Six weeks later, we took the liver and lung of mice after cervical dislocation and fixed the specimens with formaldehyde solution and embedded with paraffin. The paraffin fixed tissues were serial sectioned and stained with hematoxylin-eosin staining to identify metastastic nodules. All animal studies met the National Institutes of Health guidelines and were approved by the Committee on the Ethics of Animal Experiments of the Second Xiangya Hospital of Central South University.

### Statistical analysis

Statistical analysis was conducted using the statistical software PASW Statistics version 18.0 (SPSS, Chicago, IL, USA). We analyzed data for SYNJ2BP expression in fresh specimens with Mann-Whitney U-test, adopted Fisher’s exact test to analyze categorical data, and independent *t* test for continuous data. Spearman rank-correlation analysis was used to analyze the correlation between SYNJ2BP expression and clinicopathological features. We gained overall survival (OS) and disease-free survival (DFS) curves using the Kaplan-Meier method, and compared differences between the two groups by log-rank test. Univariate and multivariate analysis was analyzed by Cox proportional hazard regression model to verify the independent risk factors. A two-tailed *P* value of less than 0.05 was considered as statistical significance. *P* values <0.05 was considered statistically significant.

## Results

### SYNJ2BP is down-regulated in human HCC tissues and HCC cell lines

**Fig. 1:**
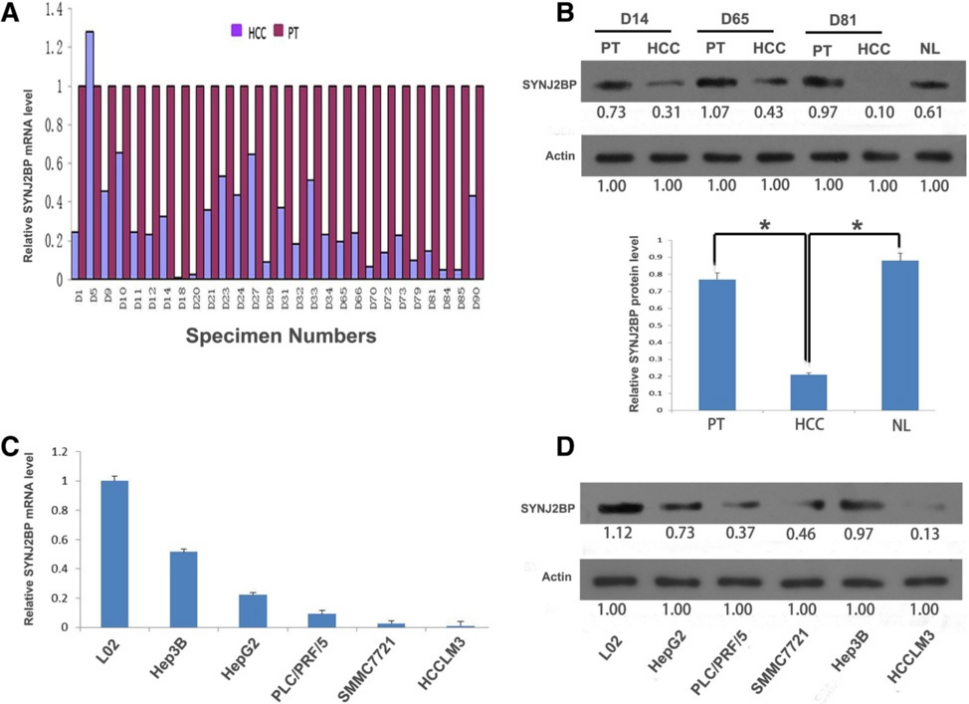
Synaptojanin 2 Binding Protein (SYNJ2BP) is frequently down-regulated in hepatocellular carcinoma(HCC) tissues and HCC cell lines. **a** The SYNJ2BP mRNA expression levels in 28 paired HCC tissues and peritumoral tissues (PTs) were obtained from quantitative real-time polymerase chain reaction (qRT-PCR). Fold inductions were calculated using the formula 2-(△△Ct), GAPDH was used as internal control. The expression of SYNJ2BP mRNA in PTs was used to normalize (value set to 1) the expression of SYNJ2BP mRNA in HCC tissues. **b** Western blot was also adopted to validate SYNJ2BP expression levels in HCC tissues and PTs. The protein expression level of SYNJ2BP in HCC tissues was significantly lower than that in PTs. *P < 0.05. But there were not significant differences between PTs and NLs (*P* = 0.442). **c, d** mRNA and protein expression levels of SYNJ2BP were detected by qRT-PCR and western blot in one normal liver cell line (L02) and 5 HCC cell lines. The results showed that compared to L02 cells, Hep3B cells had the highest SYNJ2BP expression level, while HCCLM3 cells had the lowest expression level. Data were normalized to the expression level of SYNJ2BP in L02. *P < 0.05. β-actin was used as the loading control

Our analysis showed that both SYNJ2BP mRNA and protein expressions were decreased in HCC tissues. Quantitative real-time polymerase chain reaction (qRT-PCR) results revealed that compared with peritumoral tissues (PTs), mRNA expression of SYNJ2BP in HCC tissues were significantly lower and the median fold-change was 0.23 (range, 0.01–1.28) (Fig. 1a). Similarly, western blot results showed that the expression levels of SYNJ2BP protein in HCC tissues were also significantly lower than that of PTs(0.31 ± 0.04 versus 0.83 ± 0.081; *P* < 0.01). In addition, the protein expression difference between PTs and NLs was not statistically significant (*P* > 0.05) (Fig. 1b). Meanwhile, the same techniques above were also used to detect SYNJ2BP expression in L02 and HCC cell lines with different metastatic potential [31]. The analysis showed that the mRNA expression of SYNJ2BP in L02 cells was the highest followed by Hep3B, the relative expression fold in Hep3B, HepG2, PLC/PRF/5, SMMC7721 and HCCLM3 was 0.52, 0.28, 0.15, 0.03, and 0.01 respectively; we also found that the protein expression levels of SYNJ2BP were the lowest in HCCLM3 cells which owns strong metastatic ability, the differences were statistically significant (Fig. 1c, d).

### Low expression of SYNJ2BP in HCC tissues is associated with poor clinicopathologic features of HCC

**Fig. 2:**
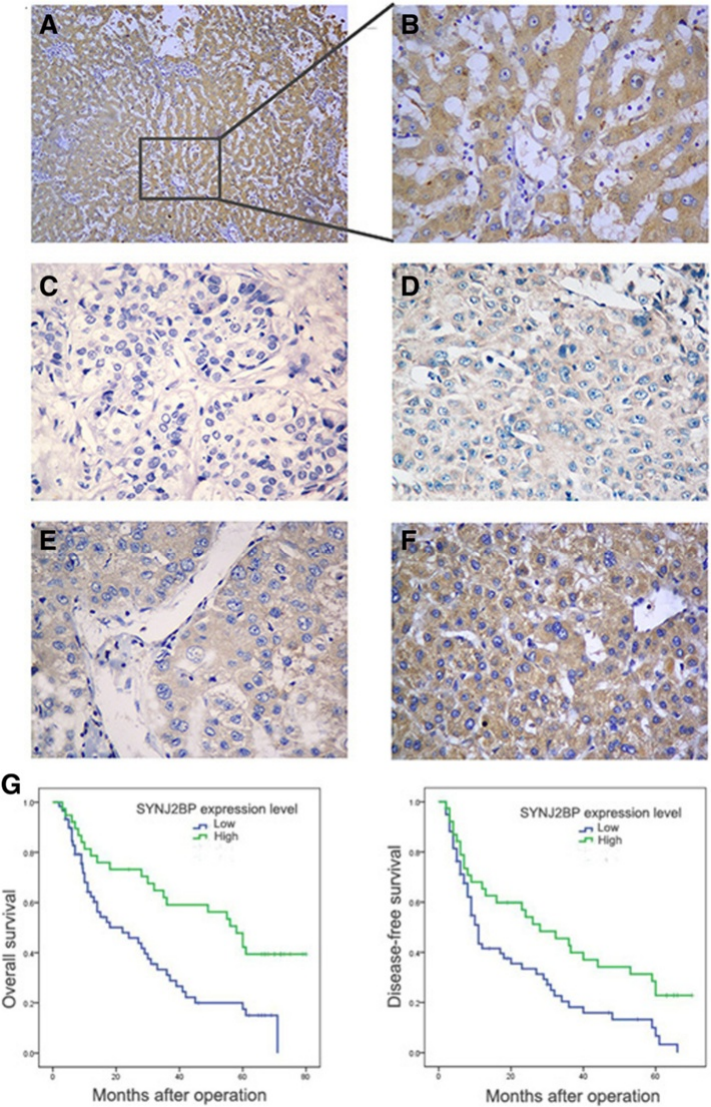
Immunohistochemistry of Synaptojanin 2 Binding Protein (SYNJ2BP) protein is down-regulated in HCC tissues and is closely related to patient survival. Here shows representative images: **a, b** SYNJ2BP expression level is strongly high in PTs. **c** SYNJ2BP expression scored as 0. **d** SYNJ2BP expression scored as 1+. **e** SYNJ2BP expression scored as 2+. **f** SYNJ2BP expression scored as 3+. Survival curves were calculated with the Kaplan-Meier method, differences were evaluated using log-rank test. **g** According to IHC results, patients were divided into low expression group (SYNJ2BP expression scored as 0 or 1+) and high expression group (SYNJ2BP expression scored as 2 or 3+). Results showed that HCC patients from the low expression group had poorer OS (*P* = 0.001) and DFS (*P* = 0.006) than those from the high expression group. Original magnification: 400× for (**b**); 100× for (**a**) and (**c** to **f**)

All the above findings suggested that SYNJ2BP probably played a role in HCC suppressing, so we thought it necessary to do further exploration. Firstly we recruited patients and their corresponding specimens mentioned above and detected SYNJ2BP expression by immunohistochemistry (IHC). IHC results showed that SYNJ2BP expression in HCC were significantly lower than that of PTs (Fig. 2a to f). According to IHC results patients were divided into SYNJ2BP high expression group (59 cases) and low expression group (39 cases). Through Chi-square test we found that SYNJ2BP low expression level was significantly associated with large tumor size(*P* = 0.002), multiple tumor nodules (*P* < 0.001), vascular invasion(*P* < 0.001), high TNM stage(*P* < 0.001) and high BCLC stage(*P* = 0.001) (Table 1), which belonged to poor clinicopathological features of HCC. These results indicated that SYNJ2BP expression was closely related to biological behavior of HCC and could probably affect the prognosis of HCC patients.

**Table 1:** Correlations between SYNJ2BP expression and clinicopathological features of HCC

		SYNJ2BP		
Clinicopathologic Variables	n	Low expression	High expression	*P* Value ^a^
Gender				
Male	84	32	52	
Female	14	7	7	0.399
Age (years)				
≤ 60	71	28	43	
> 60	27	11	16	0.906
Hepatitis B status				
Negative	17	5	12	
Positive	81	34	47	0.336
AFP(ng/ml)				
<20	28	11	17	
≥20	70	28	42	0.948
Liver cirrhosis				
Presence	81	5	12	
Absence	17	34	47	0.336
Tumor size (cm)				
≤ 3	36	7	29	
>3	62	32	30	**0.002**
Tumor nodule number				
Single	47	7	40	
Multiple (≥2)	51	32	19	**<0.001**
Vascular invasion				
Presence	53	10	43	
Absence	45	29	16	**<0.001**
Child-Pugh classification				
A	76	31	45	
B	22	8	14	0.709
TNM stage				
I	40	8	32	
II	9	2	7	
III	49	29	20	**<0.001**
BCLC stage				
0-A	54	9	45	
B	9	4	5	
C	35	26	9	**0.001**
Edmonson-Steiner grade				
I-II	72	26	46	
III-IV	26	13	13	0.215

*Abbreviations*: *AFP* alpha-fetoprotein, *TNM* tumor node metastasis, *BCLC* Barcelona Clinic Liver Cancer

^a^ Bold indicate statistical significance

### Low expression of SYNJ2BP in HCC tissues is associated with poor HCC prognosis

In order to verify the association of low SYNJ2BP expression with poor clinicopathological features of HCC, we went ahead to do clinical follow-up study on the same patients. We analyzed the association between SYNJ2BP expression level and prognosis. Survival curves revealed that patients in the low expression group had shorter OS and DFS than those in the other group. The 1-, 3- and 5-year OS of patients with low SYNJ2BP expression were 62.2, 31.1 and 17.5 %; 78.6, 59.1 and 42.2 % for the patients in high SYNJ2BP expression group. The 1-, 3-, and 5-year DFS of patients in the low SYNJ2BP expression group were 41.5, 18.1 and 6.6 %, which was significantly lower than those in the other group (65.2, 39.9 and 22.8 %) (Fig. 2g).

In the meantime, we constructed Cox proportional hazard regression model to see whether SYNJ2BP could serve as an independent risk factors for HCC prognosis. Firstly, through univariate and multivariate survival analysis,we validated that SYNJ2BP expression level(*P* = 0.002) vascular invasion(*P* < 0.001) and BCLC(*P* = 0.012) stage were independent risk factors for overall survival(OS) (Table 2). In addition, SYNJ2BP expression level (*P* = 0.030) vascular invasion (*P* = 0.031) and TNM stage (*P* = 0.011) were also considered to be independent risk factors for disease-free survival (DFS) in the same cases (Table 3). As poor prognosis of HCC is mainly due to tumor growth and metastasis, we naturally associated SYNJ2BP with HCC metastasis and growth.

**Table 2:** The Cox proportional hazard regression analyses for overall survival

Variables	n	Univariable Analysis			Multivariable Analysis
		HR (95% CI)	*P* Value ^a^	HR (95% CI)	*P* Value ^a^
Gender					
Male	14	1			
Female	84	0.820(0.390-1.727)	0.602		n.a.
Age (years)					
>60	27	1			
≤60	71	0.815(0.456-1.456)	0.490		n.a.
Hepatitis B status					
Negative	17	1			
Positive	81	0.982(0.496-1.942)	0.958		n.a.
AFP(ng/ml)					
≥20	70	1			
<20	28	0.941(0.549-1.612)	0.824		n.a.
Liver cirrhosis					
Absent	17	1			
Present	81	0.606(0.329-1.118)	0.109		n.a.
Tumor size (cm)					
≤3	36	1		1	
> 3	62	2.998(1.714-5.244)	**<0.001**	0.901(0.543-1.496)	0.687
Number of tumors					
Single	47	1		1	
Multiple (≥ 2)	51	2.772(1.632-4.707)	**<0.001**	1.004(0.479-2.106)	0.991
Vascular invasion					
Absent	45	1		1	
Present	53	2.631(1.570-4.410)	**<0.001**	1.758(1.313-2.353)	**<0.001**
Child-Pugh classification					
A	76	1			
B	22	0.862(0.459-1.617)	0.643		n.a.
Edmondson-Steiner grade					
I-II	72	1			
III-IV	26	0.984(0.573-1.688)	0.952		n.a.
TNM stage					
I/ II	49	1		1	
III	49	2.003(1.490-2.692)	**<0.001**	0.861(0.507-1.463)	0.580
BCLC					
0-A	54	1		1	
B/C	44	1.818(1.381-2.394)	**<0.001**	2.118(1.177-3.814)	**0.012**
SYNJ2BP expression					
Low	39	1		1	
High	59	0.216(0.122-0.384)	**<0.001**	0.279(0.125-0.620)	**0.002**

*Abbreviations*: *AFP* alpha-fetoprotein, *TNM* tumor node metastasis, *BCLC* Barcelona Clinic Liver Cancer, *n.a*. not adopted

^a^ Bold indicate statistical significance

**Table 3:** The Cox proportional hazard regression analysis for disease-free survival

Variables	n	Univariable Analysis			Multivariable Analysis
		HR (95% CI)	*P* Value ^a^	HR (95% CI)	*P* Value ^a^
Gender					
Male	14	1			
Female	84	0.618(0.308-1.240)	0.176		n.a.
Age (years)					
>60	27	1			
≤60	71	0.589(0.342-1.011)	0.055		n.a.
Hepatitis B status					
Negative	17	1			
Positive	81	0.649(0.367-1.145)	0.135		n.a.
AFP(ng/ml)					
≥20	70	1			
<20	28	0.719(0.448-1.156)	0.173		n.a.
Liver cirrhosis					
Absent	17	1			
Present	81	0.662(0.369-1.188)	0.167		n.a.
Tumor size (cm)					
≤3	36	1		1	
> 3	62	1.901(1.175-3.074)	**0.009**	1.663(0.921-3.004)	0.092.
Number of tumors					
Single	47	1		1	
Multiple (≥ 2)	51	1.677 (1.059-2.656)	**0.027**	0.629(0.337-1.176)	0.147
Vascular invasion					
Absent	45	1		1	
Present	53	2.633(1.625-4.268)	**<0.001**	2.023(1.679-2.998)	**0.031**
Child-Pugh classification					
A	76	1			
B	22	1.130 (0.640-1.993)	0.674		n.a.
Edmondson-Steiner grade					
I-II	72	1			
III-IV	26	1.138 (0.692-1.872)	0.611		n.a.
TNM stage					
I/ II	49	1		1	
III	49	1.882(1.440-2.461)	**<0.001**	1.953(1.163-3.279)	**0.011**
BCLC					
0-A	54	1		1	
B/C	44	1.776(1.386-2.277)	**<0.001**	0.941(0.589-1.503)	0.799
SYNJ2BP expression					
Low	39	1		1	
High	59	0.306(0.186-0.506)	**<0.001**	0.461(0.230-0.927)	**0.030**

*Abbreviations*: *AFP* alpha-fetoprotein, *TNM* tumor node metastasis, *BCLC* Barcelona Clinic Liver Cancer, *n.a*. not adopted

^a^ Bold indicate statistical significance

### SYNJ2BP inhibits HCC cells proliferation and migration *in vitro*

**Fig. 3:**
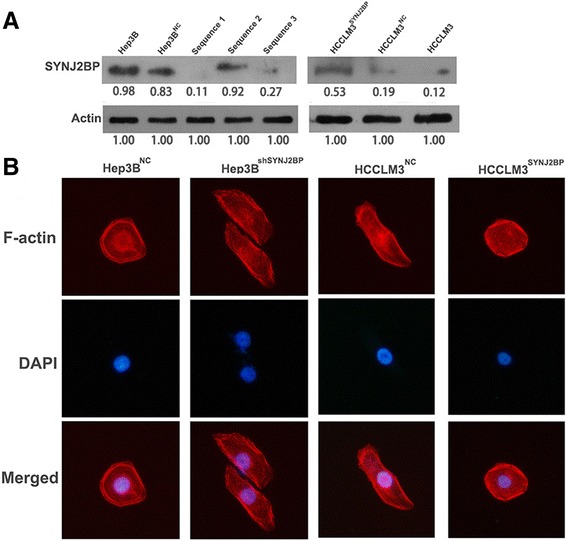
Hep3B cells were infected with SYNJ2BP lentivirus and control vector, HCCLM3 cells were also infected with shSYNJ2BP lentivirus and control vector. **a** Western blot was performed to verify transfection efficacy. Results showed that the Sequence 1 is much more effective than other two; and SYNJ2BP overexpression sequence increased expression level by more 90 %. Data were normalized to the expression level of SYNJ2BP in untreated HCC cell. **b** Representative images showed that SYNJ2BP affected the cellular morphology of HCC cells. Cell nuclei were stained with 4′,6-diamidino-2-phenylindole (blue). Original magnification: 400× for (**b**)

**Fig. 4:**
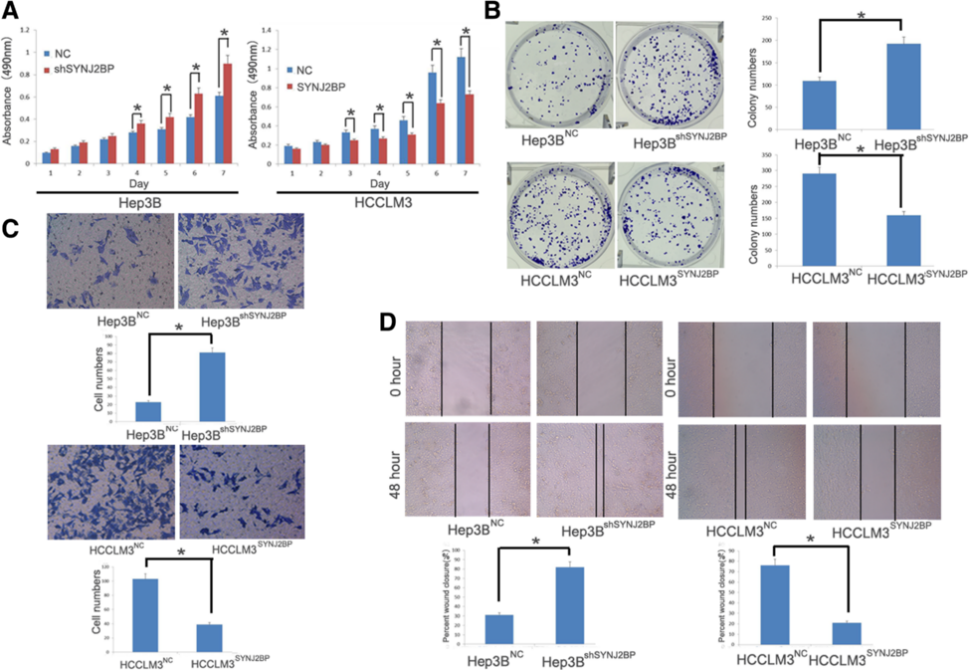
Synaptojanin 2 Binding Protein (SYNJ2BP) inhibits proliferation, invasion and migration of hepatocellular carcinoma (HCC) cells. **a** MTT assay was carried out to investigate proliferation ability in the above cells. Obvious increases of proliferation were observed in Hep3B^shSYNJ2BP^ and HCCLM3^NC^ cells compared with Hep3B^NC^ and HCCLM3^SYNJ2BP^ cells respectively. (*P* < 0.05) **b** Colony formation assay was also conducted to measure proliferation capacity of the above HCC cells. The results showed us that more colonies were formed in Hep3B^shSYNJ2BP^ (192 ± 19) and HCCLM3^NC^ (290 ± 28) cells as compared with Hep3B^NC^ (109 ± 21) and HCCLM3^SYNJ2BP^ (160 ± 17) cells. (*P* < 0.05) **c** Transwell assay showed that more Hep3B^shSYNJ2BP^ (81 ± 8) and HCCLM3^NC^ (103 ± 10)cells passed through the matrigel than Hep3B^NC^ (23 ± 3) and HCCLM3^SYNJ2BP^ (39 ± 2)cells. (*P* < 0.05) **d** For wound healing assay, the results showed that the closure of Hep3B^shSYNJ2BP^ (82 %) and HCCLM3^NC^ (76 %) cells was significantly faster than that of Hep3B^NC^ (31 %) and HCCLM3^SYNJ2BP^ (21 %) cells (*P* < 0.01). Original magnification: 200× for (**c)**; 100× for (**d)**; **b** pictures were captured by a camera. *P < 0.05

To determine whether SYNJ2BP correlates with HCC metastasis and growth, firstly we employed SYNJ2BP knockdown and ectopic expression lentiviruses and their negative control (NC) lentiviruses into Hep3B and HCCLM3 cell lines respectively. Through western blot assessing, we found that sequence 1 reduced the level of SYNJ2BP by more than 90 %, and the overexpression sequence increased SYNJ2BP expression level by more 90 % (Fig. 3a). And then we used the above cell lines for subsequent steps. In order to verify the above conjecture we first carried out a series of *in vitro* studies. Methyl thiazol tetrazolium (MTT) and colony formation assays were performed to assess SYNJ2BP function in cell proliferation (Fig. 4a, b). The results revealed that HCCLM3 cells expressing SYNJ2BP showed a lower proliferation rate and formed fewer colony numbers than control cells. However, Hep3B cells expressing shSYNJ2BP showed a higher proliferation rate and formed more colony numbers than control cells.

Meanwhile, we adopted wound healing and transwell assays to explore SYNJ2BP function in cell invasion and migration (Fig. 4c, d). As shown in Fig. 4d, Hep3B^shSYNJ2BP^ and HCCLM3^NC^ cells were more hypermigratory. HCCLM3^SYNJ2BP^ cells closed much slower than that of HCCLM3^NC^ cells (45 % versus 95 %, *P* <0.01); Hep3B^shSYNJ2BP^ cells closed much faster than that of Hep3B^NC^ cells. Meanwhile, the transwell assay showed that fewer HCCLM3^SYNJ2BP^ cells passed through the matrigel than that of HCCLM3^NC^ cells; more Hep3B^shSYNJ2BP^ cells passed through the matrigel than that of Hep3B^NC^ cells. Additionally, we also conducted immunostaining to examine morphologic changes of cells. As shown in Fig. 3B, SYNJ2BP suppressed stretch of F-actin, while knock down of SYNJ2BP leaded to formation of stress fiber-like structures, cell cytoskeletal and motility changes is closely related to tumor metastasis [32]. All in all, it is reasonable to presume that SYNJ2BP could inhibit HCC metastasis and growth.

### SYNJ2BP suppresses HCC growth and metastasis *in vivo*

**Fig. 5:**
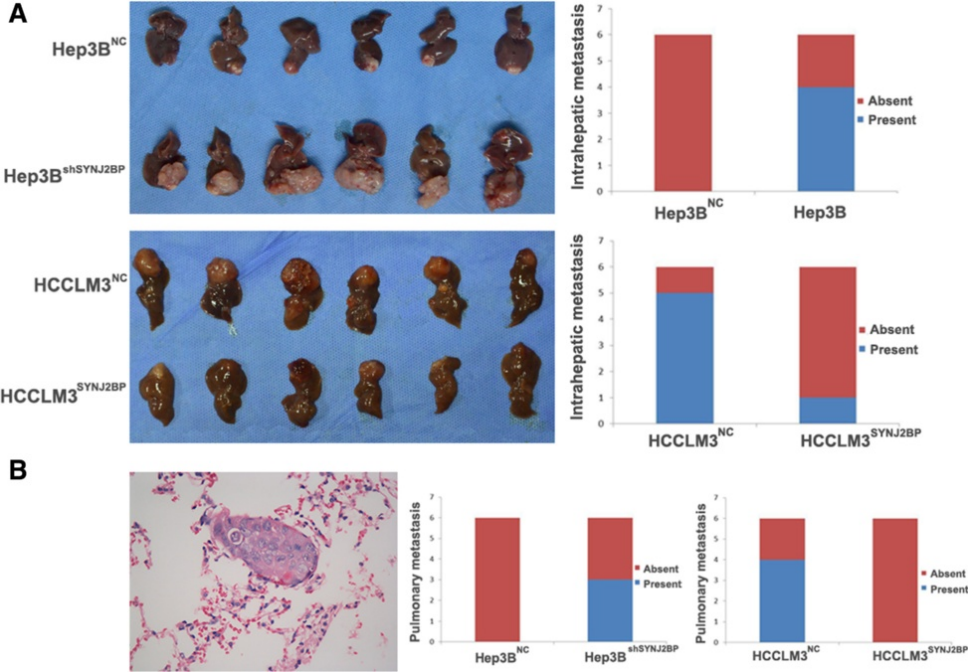
Synaptojanin 2 Binding Protein (SYNJ2BP) inhibits HCC tumor growth and metastasis *in vivo.* The nude mice model was built using Hep3B^NC^, Hep3B^shSYNJ2BP^, HCCLM3^NC^ and HCCLM3^SYNJ2BP^ cells. **a** Orthotopic tumor size in these two groups was calculated and compared. The size of tumors in Hep3B^NC^ and HCCLM3^SYNJ2BP^ groups was significantly smaller than that of Hep3B^shSYNJ2BP^ and HCCLM3^NC^ groups respectively (*P* < 0.05). **b** Serial sections of lung from model mice were stained with H&E to identify metastatic nodules, results showed that SYNJ2BP overexpression groups contained fewer(or even none) metastasis nodules than those of their corresponding groups. Original magnification: 400× for (**b**); pictures from (**a**) were captured with the aid of a canon camera

In the following research, we then developed HCC mouse models using HCC cell lines to study SYNJ2BP on HCC growth and metastasis. The results showed that SYNJ2BP inhibited subcutaneous and orthotopic tumor size significantly, as tumors originated from SYNJ2BP-transduced HCCLM3 cells were dramatically smaller than their control group; and tumor volume of the shSYNJ2BP group of Hep3B cells was greater than the control group (Fig. 5a). After harvest lungs were collected for Hematoxylin and eosin (H&E) staining, as shown in Fig. 5b, we found that SYNJ2BP overexpression groups contained fewer metastatic nodules than those of the corresponding groups. Taken together, all the above data supported that SYNJ2BP plays an important part in suppression of HCC growth and metastasis.

### SYNJ2BP exerts its function by activating DLL4 signaling

**Fig. 6:**
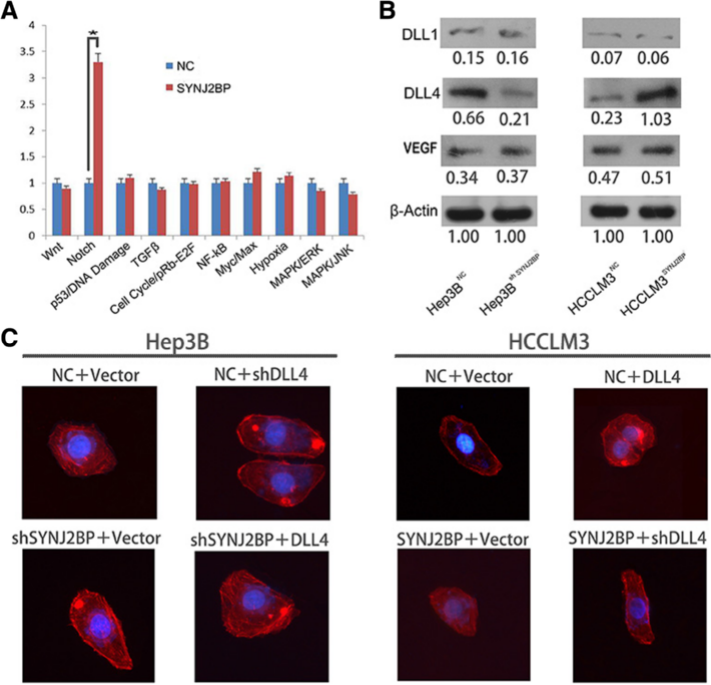
Synaptojanin 2 Binding Protein (SYNJ2BP) stimulates Notch signaling in hepatocellular carcinoma (HCC). Previous HCC cells transfected with DLL4 expression or shDLL4 or control vectors were used in these studies. **a** Notch signaling was stimulated in cells overexpressing SYNJ2BP while the other signaling pathways showed no significant change. **b** Western blot was employed to detect expression levels of DLL4, DLL1 and VEGF, only DLL4 was positively related to SYNJ2BP. **c** Representative images showed that overexpression or silencing of DLL4 mimics or abrogates SYNJ2BP mediated cellular morphology changes of HCC cells. Data were normalized to the expression level of SYNJ2BP in untreated HCC cell. Cell nuclei were stained with 4, 6-diamidino-2-phenylindole (blue). Original magnification: 400× for C. *P < 0.05

To investigate the mechanisms by which SYNJ2BP suppresses HCC growth and metastasis, we firstly adopted the Cignal Finder Cancer 10-Pathway Reporter Array to explore signaling pathways involved in this process. As shown in Fig. 6a, in SYNJ2BP overexpression cells, Notch signaling was stimulated and the activation degree is significantly higher than that of control cell (3.79 ± 0.15, *P* < 0.05). As Notch signaling is crucial to cell proliferation and migration which shows good agreement with our preliminary findings, so we thought it necessary to go on exploration tracing Notch signaling.

Previous studies have uncovered that SYNJ2BP could stabilize DLL1 and DLL4-mediated Notching signaling in endothelial cells which is particularly important for sprouting angiogenesis [16]. Because also in endothelial cells, DLL4 restrains angiogenic response triggered by vascular endothelial growth factor (VEGF) [33]. We hypothesize that SYNJ2BP sustains DLL4 to suppress tumor growth and metastasis in human HCC. To test this hypothesis, we detected expression levels of DLL1, DLL4 and VEGF in Hep3B^NC^, Hep3B^shSYNJ2BP^, HCCLM3^NC^ and HCCLM3^SYNJ2BP^ cells. The results showed that knockdown of SYNJ2BP decreased DLL4 expression level in HCC (Fig. 6b), however, there’s no expression difference of DLL1 and VEGF between Hep3B^NC^and Hep3B^shSYNJ2BP^ cells. Opposite to Hep3B cells, overexpression of SYNJ2BP in HCCLM3 cells elevated DLL4 expression but didn’t affect DLL1 and VEGF. The results showed that DLL4 was positively related to SYNJ2BP, however, DLL1 and VEGF had no correlation with SYNJ2BP.

### SYNJ2BP stabilizes DLL4 level to suppress HCC growth and metastasis

**Fig. 7:**
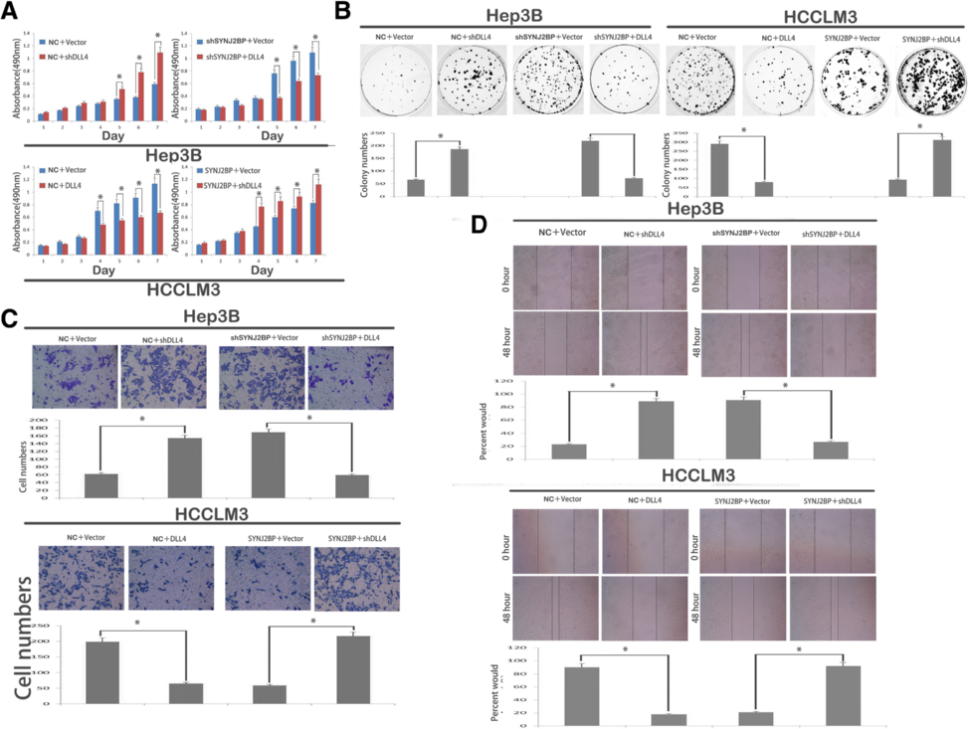
Overexpression or silencing of Delta-Like 4 (DLL4) imitates or counteracts Synaptojanin 2 Binding Protein (SYNJ2BP) mediated proliferation and invasion of Hep3B and HCCLM3 cells. DLL4 expression or shDLL4 or control vectors were transfected into previous HCC cells. The MTT (**a**) and colony formation analysis (**b**) were used to evaluate the proliferation abilities in Hep3B and HCCLM3 cells. The wound healing assay (**c**) and transwell assay (**d**) showed the migration and invasion abilities of Hep3B and HCCLM3 cells. *P < 0.05. NC: Negative Control. Original magnification: 200× for C, 100× for D; pictures from B were captured with the aid of a canon camera

To confirm whether SYNJ2BP exerts its function by sustaining DLL4, we firstly induced or silenced DLL4 expression in previous HCC cells. The following loss- and gain-of-function assay showed that overexpression of DLL4 resembled SYNJ2BP-mediated proliferation and migration of HCC cells; conversely, silencing of DLL4 blocked SYNJ2BP function (Fig. 7). Also, immunofluorescence (IF) staining showed that cell morphology changed significantly after transfection with DLL4 or short hairpin (sh) DLL4. Upon DLL4 expression, cells displayed shrinkage of F-actin; whereas knock down of DLL4 correlated with formation of stress fiber-like structures(Fig. 6c). All these results suggest that SYNJ2BP suppresses tumor growth and metastasis by targeting Notch signaling in hepatocellular carcinoma. These results confirmed that restoration of DLL4 could resemble SYNJ2BP in proliferation and migration of HCC cells, suggesting that SYNJ2BP suppresses HCC growth and metastasis through DLL4 pathway.

## Discussion

As one of the most common malignancies, HCC incidence is still rising worldwide [1]. Although there have been substantial advances in surgical and medical treatment of HCC within past decades, the mortality is remaining obstinately high [34]. Surveillance was never cost-effective as the HCC incidence was high enough. It’s not difficult to see why as recurrence and metastasis stand behind the situation above [35], and even worse, mechanisms underlying HCC recurrence still remain elusive. Through years of research work, we placed more weight on illustrating mechanisms of HCC metastasis and hope to develop effective therapeutic strategies to improve the quality of life for HCC patients.

SYNJ2BP is a protein coding gene whose encoding product is highly conserved in all eukaryotic species. There have been studies demonstrating that SYNJ2BP possesses a global role in regulating physiological processes such as sprouting angiogenesis, stabilization or localization of transmembrane proteins etc. [36] As angiogenesis plays a part in cancer [19], and in the instance of malignancy, HCC develops a neoplasm and the formation of new vessels is required for its progression [37]. On the other hand, SYNJ2BP owns a PDZ domain through which it interacts with ActRIIs to reduce activin to inhibit tumor growth [38–40]. So there may be good reason to believe SYNJ2BP is essential to human HCC. However so far, the role of SYNJ2BP in human HCC is completely unknown.

In this study, we adopted a series of techniques to validate the role of SYNJ2BP in HCC metastasis and tried to investigate the potential mechanisms. Firstly we measured SYNJ2BP expression in specimens by Real-time PCR and western blot analysis, we found that both SYNJ2BP mRNA and protein expression were significantly lower in HCC tissues than that of corresponding PTs. To confirm the findings above, we further detected SYNJ2BP expression in HCC cell lines with different invasion potential. Interestingly SYNJ2BP was down-regulated in HCC cell lines and the expression level was inversely proportional to high metastatic potential. For the reason that SYNJ2BP was down-regulated in HCC tissues and HCC cell lines, we naturally hypothesized that up-regulation of SYNJ2BP might inhibit HCC development and may act as a tumor suppressor. So secondly, we further explored whether SYNJ2BP expression correlates with HCC prognosis. Through this study we found that SYNJ2BP expression was significantly correlated with poor clinicopathological characteristics of HCC. We also proved that SYNJ2BP was associated with OS and DFS in patients with HCC and could serve as an independent risk factor. Accordingly SYNJ2BP highly likely acts as a tumor suppressor in HCC development.

Next, we continued exploration of SYNJ2BP biological function *in vitro* and *in vivo*. According to previous detection, we employed shSYNJ2BP vector to knockdown SYNJ2BP expression in Hep3B cell, and overexpression vector to elevate SYNJ2BP expression in HCCLM3 cell. Through a serial of approaches we found that ectopic SYNJ2BP expression suppressed HCC cell proliferation and metastasis potency, and down-regulation of SYNJ2BP showed an opposite effect. The *in vivo* animal model showed that overexpression of SYNJ2BP could inhibit HCC growth and metastasis, while suppression of SYNJ2BP showed opposite effects.

As Notch ligand, DLL4 is involved in angiogenesis, endothelial cell proliferation and migration and angiogenic sprouting ect [41]. And blockade of DLL4 causes endothelial disruption. There have been studies showing that in endothelial cells, SYNJ2BP could stabilize DLL1 and DLL4-mediated Notch signaling leading to poor angiogenesis [16]. However, whether or not the same mechanism exists in liver cells remains unknown yet. So, we hypothesize that SYNJ2BP suppress HCC growth and metastasis through stabilizing DLL4. To verify the above hypothesis, firstly, the Cignal Finder Cancer 10-Pathway Reporter Array was adopted to screen pathways involved, interestingly, Notch signaling was indeed activated. So next, we detected expressions of key proteins tracing Notch signaling and VEGF, results showed that only DLL4 increased accompanying elevated expression of SYNJ2BP, vise versa. Therefore, we introduced DLL4 or short hairpin shDLL4 into above HCC cells and conducted validation *in vitro.* The following loss- and gain-of-function assay showed that overexpression of DLL4 resembled SYNJ2BP-mediated proliferation and migration of HCC cells; conversely, silencing of DLL4 blocked SYNJ2BP function. Collectively, decreased expression of SYNJ2BP in HCC likely contributes to HCC growth and metastasis, partly through DLL4 pathway.

In conclusion, SYNJ2BP is decreased in HCC. We confirm that SYNJ2BP indeed possesses the potency to suppress HCC growth and metastasis through activating DLL4 pathway. This study provides, for the first time, evidence for a link between the biological activity of SYNJ2BP and HCC growth and metastasis. That means this great scientific finding, happily, unravels an unexpected function of SYNJ2BP that paves the way for early detection of HCC recurrence.

## Conclusion

Altogether, through *in vitro* and *in vivo* exploration, we uncover SYNJ2BP suppresses HCC growth and metastasis. The preliminary investigation showed that SYNJ2BP may partly exert its function through inducing DLL4 signaling pathway. However, the specific mechanism how SYNJ2BP exerts its function in HCC still requires further study. We believe with the development of modern technology there will be new findings on the way investigating HCC.

For all we know, this is the first report to associate the expression of SYNJ2BP with HCC. Our research indicates that SYNJ2BP can be used as a potential marker for HCC and may serve as a target for HCC treatment in the near future.

## Abbreviations

AFP, alpha-fetoprotein; BCLC, Barcelona Clinic Liver Cancer; DAB, diaminobenzidine; DAPI, 4′,6-diamidino-2-phenylindole; DFS, disease-free survival; DLL1, Delta-Like 1; DLL4, Delta-Like 4; H&E, hematoxylin-eosin; HBsAg, hepatitis B surface antigen; HCC, hepatocellular carcinoma; IHC, immunohistochemistry; n.a., not adopted; NC, negative control; NLs, normal live tissues; OS, overall survival; PMSF, phenylmethanesulfonyl; PTs, peritumoral tissues; PVDF, polyvinylidenefluoride; qRT-PCR, quantitative real-time reverse transcription polymerase chain reaction; SDS-PAGE, sodium dodecyl sulfate-polyacrylamide gel electrophoresis; SYNJ2BP, Synaptojanin 2 Binding Protein; TNM, tumour node metastasis classification; VEGF, Vascular Endothelial Growth Factor.
